# Cardiac functional stress imaging: A sequential approach with stress echo and cardiovascular magnetic resonance

**DOI:** 10.1186/1476-7120-5-47

**Published:** 2007-12-04

**Authors:** Rosa Sicari, Alessandro Pingitore, Giovanni Aquaro, Emilio G Pasanisi, Massimo Lombardi, Eugenio Picano

**Affiliations:** 1 CNR, Institute of Clinical Physiology, Pisa, Italy

## Abstract

**Aims:**

The aim of the study was to assess the feasibility and accuracy of an integrated stress imaging algorithm with echo first and second-line Cardiac Magnetic Resonance (CMR) in selected cases. Stress echo (SE) is widely used for non-invasive diagnosis of coronary artery disease (CAD), but difficult patients and ambiguous responses may be met even with top-level technology and expertise. CMR might ideally complement SE in well-selected cases with unfeasible and/or ambiguous and/or submaximal results.

**Methods and results:**

152 in-hospital patients with chest pain and normal baseline function were referred for SE and coronary angiography. Of the initial population, 33 were shunted to CMR due to poor acoustic window or ambiguous or submaximal SE test. The only criterion of positivity for both techniques was the presence of regional wall motion abnormalities in at least 2 contiguous segments. Coronary angiography was performed independently of test results. Significant CAD was identified by a >50% quantitatively assessed diameter reduction in at least 1 major coronary vessel.

CAD was present in 88 patients. Interpretable and diagnostic stress test were obtained in 143 patients with the sequential algorithm. The sequential (SE in 110 + CMR in 33 patients) algorithm showed a sensitivity of 76% (95% CI 66% to 85%) specificity of 87% (95% CI 76% to 95%) and accuracy of 80% (95% CI 73% to 86%).

**Conclusion:**

A sequential functional stress imaging algorithm with stress echo first and stress CMR in selected cases is feasible, clinically realistic and allows an efficient, radiation-free diagnosis of CAD.

## Background

Stress echocardiography is an established cost-effective technique for the detection of coronary artery disease [1]. According to the guidelines of European Society of Cardiology and American Society of Echocardiography – stress echocardiography (with exercise, dobutamine or dipyridamole) is a class I indication (of documented effectiveness and usefulness) for the diagnosis of coronary artery disease and for the prognostic stratification of patients with known coronary artery disease [2,3]. The widespread use in the clinical practice has become possible only after evidence collected through large scale multicenter studies that demonstrated its feasibility, safety, diagnostic and prognostic accuracy [4-9]. Its major limitation is related to a high inter-observer variability and to operator-dependent expertise that might be overcome by an appropriate training and the use of strict reading criteria [10-12]. Moreover, a percentage, although small, of patients is not feasible for echo scanning due to an acoustically hostile window (chronic obstructive pulmonary disease, conformation of the thorax etc.). Cardiac magnetic resonance (CMR) is the latest technique entering the field of cardiac imaging [13-16]. The advantages of the technique are related to its less pronounced operator-independence and the absence of ionising radiation, at the price of higher costs and lower availability when compared with echocardiography. Therefore, CMR might represent a good alternative to stress echocardiography when the latter is not feasible or test result is ambiguous. The aim of the study was to assess the feasibility and accuracy of an integrated algorithm with stress echo first and second-line stress CMR in selected cases.

## Methods

### Patients

**Figure 1:**
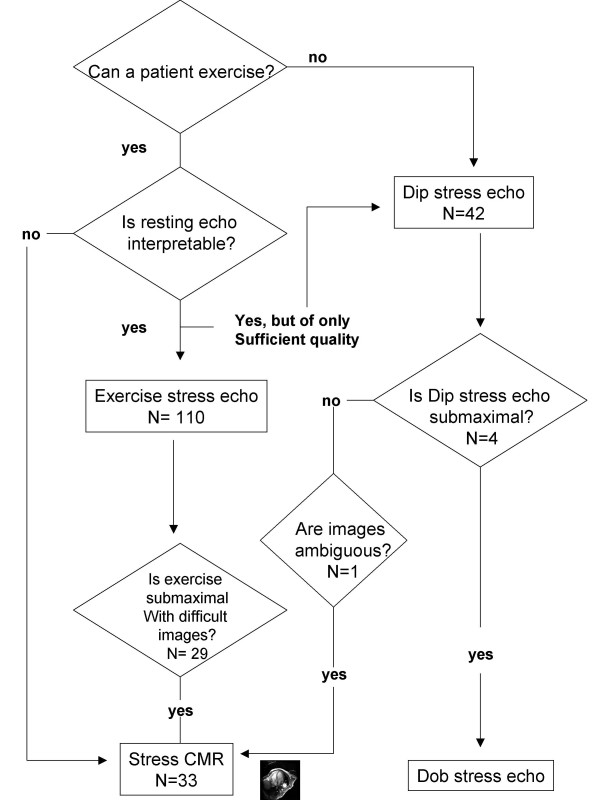
The proposed sequential algorithm.

Starting January 2003, from the data bank of the Institute of Clinical Physiology, 143 (101 males; 64 ± 9 years) non-consecutive in-hospital patients or outpatients referred to the stress echocardiography laboratory for evaluation of chest pain, or patients with known coronary artery disease, or both were enrolled in the study. Exclusion criteria were congestive heart failure, unstable angina, documented cardiomyopathy and previous myocardial infarction. All patients underwent quantitative coronary angiography independently of test result and within 1 week of testing. Of the initial population of 152 who were referred for stress echocardiography (exercise in 110 and dipyridamole in 42), 3 had a poor acoustic window, 1 had an ambiguous response, 29 had a submaximal exercise stress echo test, and in 4 dipyridamole was prematurely interrupted due to side effects (Fig. 1). Four patients who experienced side effects during dipyridamole stress echocardiography were referred to dobutamine stress echocardiography. The remaining 35 patients were referred to the magnetic resonance laboratory to undergo dipyridamole CMR stress test. Of them 1 did not fit into MR bore and 1 was not imaged because of claustrophobic reaction. The study was approved by the Institutional review board. The final patient population consisted of 143 patients who were included in the accuracy analysis. The pretest likelihood of coronary artery disease was estimated from age, sex and symptoms [17]; patients with known coronary artery disease were assigned a value of 100%. The estimated likelihood of coronary artery disease was less than 50% in 9 patients *(6%)*, 50% to 80% in 43 patients (*30%*), and greater than 80% in 91 patients (*64%*). According to individuals needs and physician's choices, 84 patients were evaluated after anti-anginal drugs had been discontinued, and 59 patients were evaluated during anti-anginal treatment (nitrates and/or calcium antagonists and/or beta-blockers). All patients gave their written informed consent when they underwent stress echocardiography. When patients signed the written informed consent they also authorized physicians to use their clinical data. Stress echo and CMR data were collected and analysed by physicians not involved in patient care.

### Stress protocols

Two-dimensional echocardiography and 12-lead electrocardiographic (ECG) monitoring were performed in combination with high dose dipyridamole (up to 0.84 mg over 6 min) or with semi-supine exercise *ECG *in accordance to well established protocols [18-20]. Cardiac magnetic resonance was performed in combination with high dose dipyridamole with the same dose used for stress echo [18,19]. During the procedure, blood pressure and ECG were recorded each minute.

### Echocardiographic analysis

Two-dimensional echocardiographic monitoring was performed throughout and up to 5 min after the end of peak stress. Two-dimensional images were recorded at baseline and at the end of each step. Regional wall motion analysis was evaluated at baseline and at peak stress with a semiquantitative assessment of a wall motion score index (WMSI), with the 17 segment model of the left ventricle, each segment ranging from 1 = normal/hyperkinetic to 4 = dyskinetic, according to the recommendations of the American Heart Association and American Society of Echocardiography [21,22]. WMSI was derived by dividing the sum of individual segment scores by the number of interpretable segments [18]. Test positivity was defined as the occurrence of at least one of the following conditions: 1) new dyssynergy in a region with normal rest function (i.e., normokinesia becoming hypokinesia, akinesia or dyskinesia) in at least two adjacent segments. Non-echocardiographic test end-points were the following: peak dipyridamole dose; 85% of target heart rate; achievement of conventional end-points (such as severe chest pain and/or diagnostic ST segment changes). The test was also stopped, in the absence of diagnostic endpoints, for one of the following reasons of constituting a submaximal, non-diagnostic test: intolerable symptoms; limiting asymptomatic side effects, consisting of: a) hypertension (systolic blood pressure >220 mmHg; diastolic blood pressure >120 mmHg); b) hypotension (relative or absolute): >30 mmHg fall of blood pressure; c) supraventricular arrhythmias: supraventricular tachycardia or atrial fibrillation; d) ventricular arrhythmias: ventricular tachycardia; frequent, polymorphous premature ventricular beats.

### Magnetic resonance analysis

CMR has been performed on a whole body MR scanner (Signa Cvi, GE, USA), operating at 1.5T by using a dedicated cardiac 8 elements phased-array coil. CMR acquisition has been performed according to a standardized protocol. Cardiac images have been obtained using a breath-hold segmented gradient echo Steady State Free Precession (SSFP) electrocardiographic triggered sequences. The echo time was 1.7 ms; repetition time 4.0 ms; slice thickness 8 mm with no interslice gap; field of view from 320 to 380 mm; data matrix size 256 × 224; phase of field of view 0.75; trigger delay was minimum; and views per segment 8 to 12 according to heart rate; flip angle 45°. Thirty cine frames were obtained for each slice. Three short axes slices, respectively at basal, middle and distal levels and 4-, 2-, and 3-chamber views of the left ventricle (LV), have been used for calculating regional wall motion (WM). The basal middle and distal segments were defined in relation to the papillary muscles; basal segments were below and middle segments were above the appearance of papillary muscles; middle segments were in the presence of papillary muscles. The LV has been divided into a 17-segment model. WM has been semi quantitatively assessed as follows: 1 = normokinetic, 2 = hypokinetic, 3 = akinetic and 4 = dyskinetic [21]. A dipyridamole test has been considered to be positive for functional criteria when wall motion worsened in 2 contiguous myocardial segments. There were 2 experienced observers reading wall motion, who were blinded to each other and to angiographic results.

### Coronary angiography

Coronary angiography in multiple views was performed according to the standard Judkins or Sones technique. At least five views (including two orthogonal views) were acquired for the left and at least two orthogonal views for the right coronary arteries, respectively. Additional appropriate projections were obtained in case of superimposition of side branches or foreshortening of the segment of interest. A vessel was considered to have significant obstruction if its diameter was narrowed by 50% with respect to the prestenosic segment. All stenotic segments were evaluated by an automated edge detection system providing the percent stenosis diameter.

### Statistical analysis

Values are expressed as mean ± standard deviation. Proportions were compared by the chi-square statistic; a Fisher's exact test was used when appropriate. A p value <0.05 was considered statistically significant. Calculations of sensitivity, specificity and accuracy were performed according to standard definitions. The 95% CIs were calculated for each technique, and the individual intervals were compared. Differences between techniques were considered significant at the 0.05 level when 95% CI did not overlap.

## Results

The main clinical data are reported in Table 1.

**Table 1:** Clinical characteristics of study population

No. of patients	143
Age (years)	64 ± 9
Sex (male:female)	101/42
Family history	74 (52%)
Smoking habit	56 (39%)
Hypertension	90 (63%)
Diabetes	33 (23%)
Hypercholesterolemia	107 (75%)
History of angina	98 (67%)
Patients with CAD	88
1-vessel disease	31
2-vessel disease	33
3-vessel disease	24

CAD= coronary artery disease

### Coronary angiographic results

Coronary angiography demonstrated absence or only non-significant coronary artery disease in 55 patients and significant coronary artery disease in 88: Of these 31 had one vessel disease, 33 two vessel and 24 three vessel disease; left main coronary artery disease was found in 4 patients.

### Stress Echocardiography results

Stress echocardiography was diagnostic in 110 patients. Three patients had a poor acoustic window (additional file 1), 1 had an ambiguous response, 29 had a submaximal exercise stress test, and in 4 dipyridamole was prematurely interrupted due to side effects and were referred to dobutamine stress echocardiography (fig. 1). Rest WMSI was 1.0 and peak WMSI was 1.2 ± 0.3. The test was positive in 60 (54.5%) (additional files 2 and 3). Diagnostic ECG changes and chest pain were present during the test in 43 (72%) and 26 (43%) patients of the 60 with a positive test result.

### Stress CMR results

The subgroup of 35 patients with a non-diagnostic and/or suboptimal stress echocardiography underwent dipyridamole CMR. The test was feasible in 33 of them (1 did not fit into the MR bore and 1 was not imaged because of anxiety) (fig. 1). Rest WMSI was 1.0 and peak WMSI was 1.12 ± 0.2. The test was positive in 14 (42%) patients (additional files 4 and 5).

### Correlation between angiographic data and stress results

**Figure 2:**
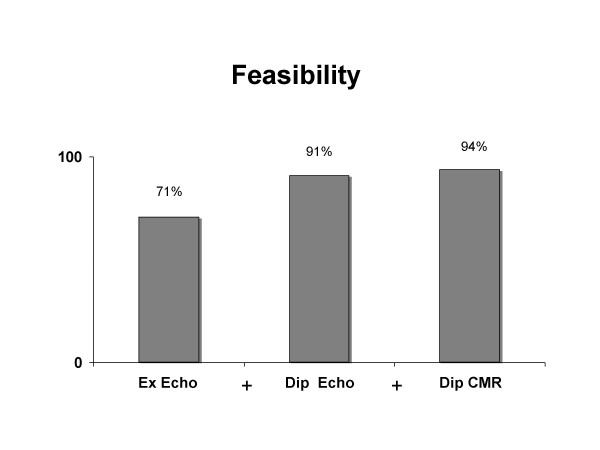
Bar graph showing the number of maximal and/or interpretable tests for each technique employed (exercise stress echo, dipyridamole stress echo and dipyridamole CMR). The number of maximal tests increases when the sequential algorithm is applied.

**Figure 3:**
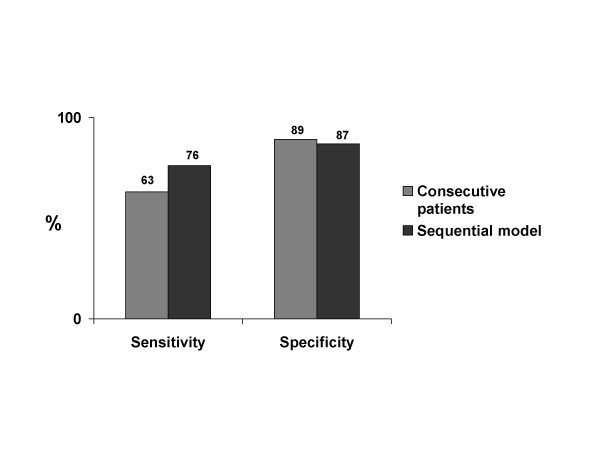
Bar graph showing sensitivity and specificity in consecutive patients and after the sequential model with CMR as a second line stress technique has been employed. Sensitivity significantly increases with only a negligible loss of specificity when the sequential model is applied.

Significant coronary artery disease was present in 71 of the 110 who underwent stress echocardiography and in 17 of the 33 who underwent dipyridamole CMR. The number of interpretable and/or maximal tests and test sensitivity significantly increased, with only a negligible loss of specificity when the sequential model was applied (fig.2 and 3). In fact sensitivity for detecting CAD was 63% (95% CI 52% to 73%) for stress echo consecutive patients and increased to 76% (95% CI 66% to 84%) for the sequential model with stress CMR; specificity was 89% (95% CI 78% to 96%) and 87% for stress CMR (95% CI 75% to 94%), respectively. Accuracy was 73% for stress echo (95% CI 65% to 80%) and increased to 80% (95% CI 73% to 86%) when stress CMR was employed.

## Discussion

Stress CMR can be used to diagnose inducible ischemia in patients unable to undergo stress echocardiography. Diagnostic accuracies of the two techniques are comparable and the more demanding and expensive CMR can be used as a second line technique only when stress echocardiography is submaximal, inconclusive or unfeasible.

In fact, image quality is one of the major limitations of stress echocardiography interpretation and this affects significantly diagnostic accuracy [12] and wall motion analysis during CMR can overcome this limitation.

### Comparison with previous studies

The results of our study are broadly consistent with several studies suggesting that both stress echo (with exercise or pharmacologic stress, both with dipyridamole and dobutamine) and CMR are excellent options for the diagnosis of CAD [23-27]. This has also been stated in recent ESC guidelines [2] and is in agreement with the recent ESC panel report on the clinical use of CMR that consider stress MR a class II examination for the diagnosis of coronary artery disease [28]. Overall, the feasibility and diagnostic accuracy of each test is lower than previously reported, but this is unavoidable when one moves from initial feasibility studies [15,26,29-31] to clinical studies where the technique is deployed in the field on consecutive, challenging and often "difficult" patients [8]. The low sensitivity of the test may be due to several factors: All patients had a normal baseline function; a high number of patients were studied under anti-ischemic therapy (59 out of 152), which reduces sensitivity of wall motion; 31 out of 88 patients had a single vessel disease; Moreover we analysed only regional wall motion for both SE and CMR, without addition of myocardial perfusion or **Coronary flow reserve** criteria. Nevertheless, the overall performance of the algorithm seemed acceptable in the "real life" context.

### Clinical implications: "nobody is perfect"

There are several tests and strategies for the evaluation of patients with known or suspected coronary artery disease, but no single strategy has been demonstrated to be superior overall. The clinical plausibility of this algorithm stems from the obvious fact that "nobody is perfect", also among stress tests, and there is the primary need to optimise economic and technological resources without reducing the standard of diagnostic excellence. In the present study a clinical practical algorithm in which stress echocardiography and stress CMR can be used in a sequential way (see fig.1) for the assessment of CAD is proposed. The 3 major aspects of the algorithm are: 1. restrict the indication to more expensive methods to patients really in need; 2 – to have a maximal result in all patients, since submaximal stress test have low diagnostic and prognostic value [19,32]; 3 – minimize long term risks due to the use of radiation [33,34] with a radiation-free stress imaging algorithm allowing to select for coronary angiography and revascularization only patients with a functionally significant, and therefore prognostically malignant, forms of coronary artery disease, who are more likely to benefit from a physiologic driven revascularization. In this algorithm, CMR replaces cardiac stress scintigraphy which gives a dose exposure corresponding to 500 (with Sestamibi) to 1,600 (with Thallium or dual isotope scan) chest x-rays per each exam [33-36]. Since 10 million cardiac stress scintigraphies are performed each year in US, the positive impact of our proposed algorhythm on downstream risks would be remarkable [37,38]

## Conclusion

A sequential functional stress imaging algorithm with stress echo first and stress CMR in selected cases is feasible, clinically realistic and allows a highly efficient, radiation-free diagnosis of CAD in almost all patients in whom cardiac stress imaging is clinically indicated.

Stress CMR is an excellent option when stress echocardiography is inconclusive or unfeasible.

## Authors' contributions

RS drafted the manuscript and performed the statistical analysis

AP, GQ, EMP, ML: acquired the data

EP: conceived the study and approved the final manuscript

All authors read and approved the final manuscript

## Supplementary Material

Additional file 1 4-chamber view of a bad acoustic window unfeasible for stress echocardiography. Click here for file

Additional file 2 High dose dipyridamole stress echocardiography. Four chamber view at rest (no wall motion abnormality is present at rest) Click here for file

Additional file 3 High dose dipyridamole stress echocardiography. Four chamber view at peak dipyridamole stress (wall motion abnormality of the apex and distal septum). Click here for file

Additional file 4 High dose dipyridamole CMR. Four chamber view at rest (no wall motion abnormality is present at rest) Click here for file

Additional file 5 High dose dipyridamole CMR. Four chamber view at peak dipyridamole stress (wall motion abnormality of the apex and distal septum). Click here for file

## References

[B1] Picano E (1992). Stress echocardiography. From pathophysiological toy to diagnostic tool. Circulation.

[B2] Fox K, Garcia MA, Ardissino D, Buszman P, Camici PG, Crea F, Daly C, De Backer G, Hjemdahl P, Lopez-Sendon J, Marco J, Morais J, Pepper J, Sechtem U, Simoons M, Thygesen K, Priori SG, Blanc JJ, Budaj A, Camm J, Dean V, Deckers J, Dickstein K, Lekakis J, McGregor K, Metra M, Morais J, Osterspey A, Tamargo J, Zamorano JL (2006). Task Force on the Management of Stable Angina Pectoris of the European Society of Cardiology, ESC Committee for Practice Guidelines (CPG). Task Force on the Management of Stable Angina Pectoris of the European Society of Cardiology, ESC Committee for Practice Guidelines (CPG). Guidelines on the management of stable angina pectoris: executive summary: the Task Force on the Management of Stable Angina Pectoris of the European Society of Cardiology. Eur Heart J.

[B3] Pellikka PA, Nagueh SF, Elhendy AA, Elhendy CA, Sawada  SG (2007). American Society of  Echocardiography recommendations for performance, interpretation, and  application of stress echocardiography.. J Am Soc Echocardiogr.

[B4] Picano E, Marini C, Pirelli S, Maffei S, Bolognese L, Chiriatti G, Chiarella F, Orlandini A, Seveso G, Colosso MQ, on behalf of the EPIC Study Group (1992). Safety of intravenous high-dose dipyridamole echocardiography. Am J Cardiol.

[B5] Picano E, Mathias W, Pingitore A, Bigi R, Previtali M, on behalf of the EDIC Study Group (1994). Safety and tolerability of dobutamine-atropine stress echocardiography: a prospective, large scale, multicenter trial. Lancet.

[B6] Picano E, Landi P, Bolognese L, Chiaranda G, Chiarella F, Seveso G, Sclavo MG, Gandolfo N, Previtali M, Orlandini A, on behalf of the EPIC Study Group (1993). Prognostic value of dipyridamole-echocardiography early after uncomplicated myocardial infarction: a large scale multicenter trial. Am J Med.

[B7] Sicari R, Pasanisi E, Venneri L, Landi P, Cortigiani L, Picano E (2003). Echo Persantine International Cooperative (EPIC) Study Group; Echo Dobutamine International Cooperative (EDIC) Study Group. Stress echo results predict mortality: a large scale multicenter prospective international study. J Am Coll Cardiol.

[B8] Pingitore A, Picano E, Varga A, Gigli G, Cortigiani L, Previtali M, Minardi G, Colosso MQ, Lowenstein J, Mathias W, Landi P (1999). Prognostic value of pharmacological stress echocardiography in patients with known or suspected coronary artery disease: a prospective, large scale, multicenter, head-to-head comparison between dipyridamole and dobutamine test. J Am Coll Cardiol.

[B9] Marwick TH, Case C, Sawada S, Rimmerman C, Brenneman P, Kovacs R, Short L, Lauer M (2001). Prediction of mortality using dobutamine echocardiography. J Am Coll Cardiol.

[B10] Picano E, Lattanzi F, Orlandini A, Marini C, L'Abbate A (1991). Stress echocardiography and the human factor: the importance of being expert. J Am Coll Cardiol.

[B11] Imran MB, Palinkas A, Pasanisi EM, De Nes M, Picano E (2002). Optimal reading criteria in stress echocardiography. Am J Cardiol.

[B12] Hoffmann R, Lethen H, Marwick T, Arnese M, Fioretti P, Pingitore A, Picano E, Buck T, Erbel R, Flachskampf FA, Hanrath P (1996). Analysis of interinstitutional observer agreement in interpretation of dobutamine stress echocardiograms. J Am Coll Cardiol.

[B13] Kim WY, Danias PG, Stuber M, Flamm SD, Plein S, Nagel E, Langerak SE, Weber OM, Pedersen EM, Schmidt M, Botnar RM, Manning WJ (2001). Coronary magnetic resonance angiography for the detection of coronary stenoses. N Engl J Med.

[B14] Kim RJ, Wu E, Rafael A, Chen EL, Parker MA, Simonetti O, Klocke FJ, Bonow RO, Judd RM (2000). The use of contrast-enhanced magnetic resonance imaging to identify reversible myocardial dysfunction. N Engl J Med.

[B15] Nagel E, Lehmkuhl HB, Bocksch W, Klein C, Vogel U, Frantz E, Ellmer A, Dreysse S, Fleck E (1999). Noninvasive diagnosis of ischemia-induced wall motion abnormalities with the use of high-dose dobutamine stress MRI: comparison with dobutamine stress echocardiography. Circulation.

[B16] Nagel E, Klein C, Paetsch I, Hettwer S, Schnackenburg B, Wegscheider K, Fleck E (2003). Magnetic resonance perfusion measurements for the noninvasive detection of coronary artery disease. Circulation.

[B17] Diamond GA, Forrester JS (1979). Analysis of probability as an aid in the clinical diagnosis of coronary artery disease. N Engl J Med.

[B18] Armstrong WF, Pellikka PA, Ryan T, Crouse L, Zoghbi WA (1998). Stress echocardiography: recommendations for performance and interpretation of stress echocardiography. Stress Echocardiography Task Force of the Nomenclature and Standards Committee of the American Society of Echocardiography. J Am Soc Echocardiogr.

[B19] Dal Porto R, Faletra F, Picano E, Pirelli S, Moreo A, Varga A (2001). Safety, feasibility, and diagnostic accuracy of accelerated high-dose dipyridamole stress echocardiography. Am J Cardiol.

[B20] Gibbons RJ, Balady GJ, Bricker JT, Chaitman BR, Fletcher GF, Froelicher VF, Mark DB, McCallister BD, Mooss AN, O'Reilly MG, Winters WL, Gibbons RJ, Antman EM, Alpert JS, Faxon DP, Fuster V, Gregoratos G, Hiratzka LF, Jacobs AK, Russell RO, Smith SC (2002). American College of Cardiology/American Heart Association Task Force on Practice Guidelines. Committee to Update the 1997 Exercise Testing Guidelines. American College of Cardiology/American Heart Association Task Force on Practice Guidelines, Committee to Update the 1997 Exercise Testing Guidelines, ACC/AHA 2002 guideline update for exercise testing: summary article: A report of the American College of Cardiology/American Heart Association Task Force on Practice Guidelines (Committee to Update the 1997 Exercise Testing Guidelines). A report of the American College of Cardiology/American Heart Association Task Force on Practice Guidelines (Committee to Update the 1997 Exercise Testing Guidelines). J Am Coll Cardiol.

[B21] Cerqueira MD, Weissman NJ, Dilsizian V, Jacobs AK, Kaul S, Laskey WK, Pennell DJ, Rumberger JA, Ryan T, Verani MS (2002). American Heart Association Writing Group on Myocardial Segmentation and Registration for Cardiac Imaging. Standardized myocardial segmentation and nomenclature for tomographic imaging of the heart: a statement for healthcare professionals from the Cardiac Imaging Committee of the Council on Clinical Cardiology of the American Heart Association. Circulation.

[B22] Lang RM, Bierig M, Devereux RB, Flachskampf FA, Foster E, Pellikka PA, Picard MH, Roman MJ, Seward J, Shanewise J, Solomon S, Spencer KT, St John Sutton M, Stewart W (2005). Recommendations for chamber quantification: a report from the American Society of Echocardiography's Guidelines and Standards Committee and the Chamber Quantification Writing Group, developed in conjunction with the European Association of Echocardiography, a branch of the European Society of Cardiology. J Am Soc Echocardiogr.

[B23] Pennell DJ, Underwood SR, Manzara CC, Swanton RH, Walker JM, Ell PJ, Longmore DB (1992). Magnetic resonance imaging during dobutamine stress in coronary artery disease. Am J Cardiol.

[B24] Baer FM, Voth E, Theissen P, Schicha H, Sechtem U (1994). Gradient-echo magnetic resonance imaging during incremental dobutamine infusion for the localization of coronary artery stenoses. Eur Heart J.

[B25] van Rugge FP, van der Wall EE, Spanjersberg SJ, de Roos A, Matheijssen NA, Zwinderman AH, van Dijkman PR, Reiber JH, Bruschke AV (1994). Magnetic resonance imaging during dobutamine stress for detection and localization of coronary artery disease. Quantitative wall motion analysis using a modification of the centerline method. Circulation.

[B26] Hundley WG, Hamilton CA, Thomas MS, Herrington DM, Salido TB, Kitzman DW, Little WC, Link KM (1999). Utility of fast cine magnetic resonance imaging and display for the detection of myocardial ischemia in patients not well suited for second harmonic stress echocardiography. Circulation.

[B27] Pingitore A, Scattini B, De Marchi D, Acquaro G, Positano V, Deiana M, Lombardi M, Picano E (2007). Head-to-head comparison between perfusion and wall motion during high dose dipyridamole magnetic resonance for the detection of coronary artery disease. Am J Cardiol.

[B28] Pennell DJ, Sechtem UP, Higgins CB, Manning WJ, Pohost GM, Rademakers FE, van Rossum AC, Shaw LJ, Yucel EK (2004). Society for Cardiovascular Magnetic Resonance Working Group on Cardiovascular Magnetic Resonance of the European Society of Cardiology. Clinical indications for cardiovascular magnetic resonance (CMR): Consensus Panel report European Heart Journal.

[B29] Picano E, Lattanzi F, Masini M, Distante A, L'Abbate A (1988). Usefulness of the dipyridamole-exercise echocardiography test for diagnosis of coronary artery disease. Am J Cardiol.

[B30] Robertson WS, Feigenbaum H, Armstrong WF, Dillon JC, O'Donnell J, McHenry PW (1983). Exercise echocardiography: a clinically practical addition in the evaluation of coronary artery disease. J Am Coll Cardiol.

[B31] McNeill AJ, Fioretti PM, el-Said SM, Salustri A, Forster T, Roelandt JR (1992). Enhanced sensitivity for detection of coronary artery disease by addition of atropine to dobutamine stress echocardiography. Am J Cardiol.

[B32] Sicari R, Cortigiani L, Bigi R, Landi PBSc, Raciti M, Picano E (2004). The Prognostic value of pharmacological stress echo is affected by concomitant anti-ischemic therapy at the time of testing. Circulation.

[B33] Picano E (2004). Informed consent and communication of risk from radiological and nuclear medicine examinations: how to escape from a communication inferno. BMJ.

[B34] Picano E (2005). Economic and biological costs of cardiac imaging. Cardiovasc Ultrasound.

[B35] Thompson RC, Cullom SJ (2006). Issues regarding radiation dosage of cardiac nuclear and radiography procedures. J Nucl Cardiol.

[B36] Einstein AJ, Moser KW, Thompson RC, Cerqueira MD, Henzlova MJ (2007). Radiation dose to patients from cardiac diagnostic imaging. Circulation.

[B37] Picano E (2003). Stress echocardiography: a historical perspective. Am J Med.

[B38] Picano E, Vano E, Semelka R, Regulla D (2007). The American College of Radiology white paper on radiation dose in medicine: deep impact on the practice of cardiovascular imaging. Cardiovasc Ultrasound.

